# Transcriptomics of dorso-ventral axis determination in *Xenopus tropicalis*

**DOI:** 10.1016/j.ydbio.2018.04.022

**Published:** 2018-07-15

**Authors:** Rita S. Monteiro, George E. Gentsch, James C. Smith

**Affiliations:** The Francis Crick Institute, Developmental Biology Laboratory, 1 Midland Road, London NW1 1AT, United Kingdom

**Keywords:** *Xenopus*, UV, Lithium, Wnt, β-catenin, Dorso-ventral, Neural induction, Cell fate, Transcriptome, RNF220

## Abstract

Amphibian embryos provide a powerful system to study early cell fate determination because their eggs are externally fertilised, large, and easy to manipulate. Ultraviolet (UV) or lithium chloride (LiCl) treatment are classic embryonic manipulations frequently used to perturb specification of the dorso-ventral (DV) axis by affecting the stability of the maternal Wnt mediator β-catenin. Such treatments result in the formation of so-called ventralised or dorsalised embryos. Although these phenotypes have been well described with respect to their morphology and some aspects of gene expression, their whole transcriptomes have never been systematically characterised and compared. Here we show that at the early gastrula stage UV-treated embryos are transcriptionally more closely related to untreated embryos than to LiCl-treated embryos. Transcriptional comparisons with dissected ventral and dorsal regions of unperturbed gastrula embryos indicate that UV and LiCl treatments indeed enrich for ventral and dorsal cells, respectively. However, these treatments also affect the balance of neural induction in the ectodermal germ layer, with LiCl stimulating pro-neural BMP inhibition and UV preferentially generating epidermis because of elevated BMP levels. Thus the transcriptomes of UV- and LiCl-treated embryos can best be described as ventro-epidermalised and dorso-neuralised. These descriptions notwithstanding, our profiling reveals several hitherto uncharacterized genes with differential expression along the DV axis. At least one of these genes, a RNF220-like ubiquitin ligase, is activated dorsally by β-catenin. Our analysis of UV/LiCl-mediated axis perturbation will enhance the mechanistic understanding of DV axis determination in vertebrates.

## Highlights

• We perturb DV axis specification in *Xenopus tropicalis* by UV or LiCl treatment.
• Transcriptome reveals that UV-treated embryos are more similar to untreated embryos.
• Comparison with ventral and dorsal cells confirms the effects of LiCl and UV in DV axis perturbation.
• We identify a RNF220-like ubiquitin ligase as a dorsal marker regulated by β-catenin.

## 1 Introduction

Amphibian eggs have an animal–vegetal axis that is defined by the differential deposition of various proteins and RNA transcripts during oogenesis. This initial radial symmetry is broken immediately after fertilisation along the prospective dorso-ventral (DV) axis. At the sperm entry point (SEP) the paternal centriole initiates microtubule polymerisation which enables a 30° rotation of the cortical layer relative to the cytoplasmic core (Elinson, 1975; Elinson and Manes, 1978; Kao and Elinson, 1988; Manes and Elinson, 1980; Vincent et al., 1986). This causes the so-called grey crescent to appear opposite the SEP, which marks the prospective dorsal side of the embryo.

The disruption of microtubule polymerisation provides a powerful approach to investigate DV axis determination (Kao and Elinson, 1988). The method of choice has frequently been the ultraviolet (UV) irradiation of the vegetal pole immediately after fertilisation. Without intact vegetal-cortical microtubules, various components of the canonical Wnt signalling pathway such as Dishevelled (Dsh), Wnt11 and GSK3-binding protein (GBP) fail to be transferred from the vegetal pole to the prospective dorsal side (Miller et al., 1999, Tao et al., 2005, Weaver et al., 2003). Consequently, the Wnt mediator β-catenin remains unstable and does not accumulate in nuclei, where it normally triggers zygotic gene expression and so regulates DV patterning (Larabell et al., 1997).

In contrast to UV irradiation, lithium chloride (LiCl) is frequently used to promote dorsal specification. LiCl stabilises β-catenin by inhibiting GSK3β, which is ubiquitously expressed and belongs to the destruction complex of the canonical Wnt pathway (Klein and Melton, 1996, Stambolic et al., 1996). The phenotypic effects of LiCl treatment on amphibian embryos depend on dosage and stage of administration (Hall, 1942, Pasteels, 1945). Addition of LiCl to 0.3 M to the medium of embryos at the 32-cell stage generates truncated tadpoles with enlarged dorso-anterior structures (Kao et al., 1986). By the 32-cell stage, the activity of canonical Wnt is normally restricted to the dorsal side of the embryo due to Dsh-mediated GSK3β inhibition; widespread LiCl-mediated inhibition of GSK3β causes ventral cells to accumulate nuclear β-catenin and thus to adopt a dorsal fate.

The degree of ventralisation and dorsalisation can be classified morphologically according to the dorso-anterior index (DAI), which ranges from 0 to 10 (Kao and Elinson, 1988): Hyperdorsalised embryos are scored as 10; normal embryos as 5; and hyperventralised embryos resembling a belly piece with no dorso-anterior structures as 0.

Here we use UV and LiCl treatment to study DV axis determination on a transcriptome-wide scale. The genomes of the allotetraploid *X. laevis* and the diploid *X. tropicalis* have been sequenced (Hellsten et al., 2010, Session et al., 2016), and this has allowed thorough analyses of whole transcriptomes in time (Collart et al., 2014, Owens et al., 2016) and space (Blitz et al., 2017, De Domenico et al., 2015; Ding et al., 2017a; Popov et al., 2017). Our comparison with the transcriptomes of dorsal- and ventral embryonic regions confirms that UV or LiCl treatments confer ventral or dorsal cell fate specification, respectively. However, these treatments also affect the balance of neural induction in the ectodermal germ layer, prompting us to characterise UV- and LiCl-treated embryos as ventro-epidermalised and dorso-neuralised. This revised characterisation of UV and LiCl phenotypes notwithstanding, we have identified some hitherto uncharacterized genes with differential expression along the DV axis. We show that a gene coding for a novel RN220-like ubiquitin ligase is directly activated by β-catenin in dorsal cells.

## 2 Results

### 2.1 UV and LiCl treatments perturb DV axis formation in *X. tropicalis*

**Fig. 1:**
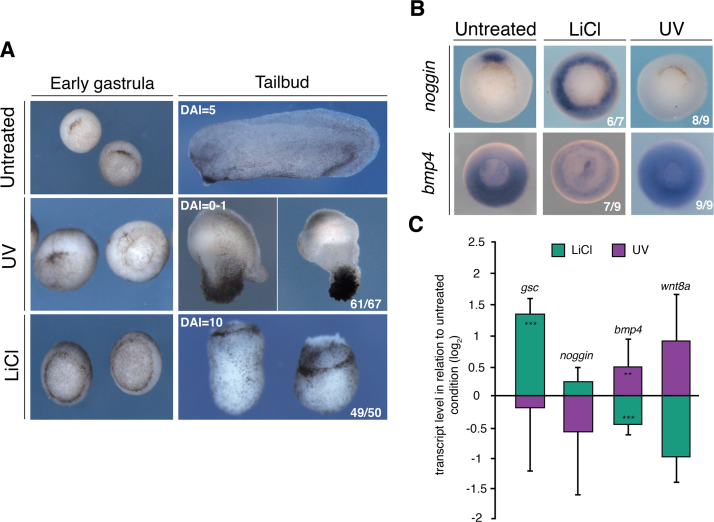
**Optimised LiCl and UV treatments of*****X. tropicalis*****embryos perturb their DV axis determination in line with the original experiments in*****X. laevis***. (A-C) DV perturbations in *X. tropicalis* embryos caused by LiCl or UV treatments. (A) Phenotypical morphologies. (B) WMISH of dorsal (*noggin*) and ventral (*bmp4*) markers in mid-gastrula embryos. (C) RT-qPCR of dorsal (*gsc* and *noggin*) and ventral (*bmp4* and *wnt8a*) markers in mid-gastrula embryos. Error bars, standard error of the mean (SEM) of biological triplicates. Student's two-tailed *t*-test: *, p < 0.1; **, p < 0.05; and ***, p < 0.01. DAI, dorso-anterior index.

The conditions required to ventralise or dorsalise *X. tropicalis* embryos were determined empirically starting with those that had been optimised for *X. laevis* (Sive et al., 1999). Hyperventralisation was achieved by irradiating the vegetal pole with shortwave UV for 2 min at a range of 2 cm from the light source within 30 min after fertilisation. This treatment often caused the blastopore lip to develop abnormally: the initial appearance of the blastopore lip was delayed, and it formed circumferentially rather than on the dorsal side. By the tailbud stage, these embryos appeared as amorphic tissue lumps known as belly pieces (DAI = 0–1) (Fig. 1A). Dorsalisation was most effective when 32-cell embryos were incubated in 0.3 M LiCl for 5 min. Hyperdorsalisation caused blastopore lip formation to be initiated on time but circumferentially (Fig. 1A). At the tailbud stage, hyperdorsalised embryos consisted of radially symmetric, extended anterior structures such as a circular cement gland at the expense of any posterior structures such as trunk and tail (DAI = 10).

**Fig. S1:**
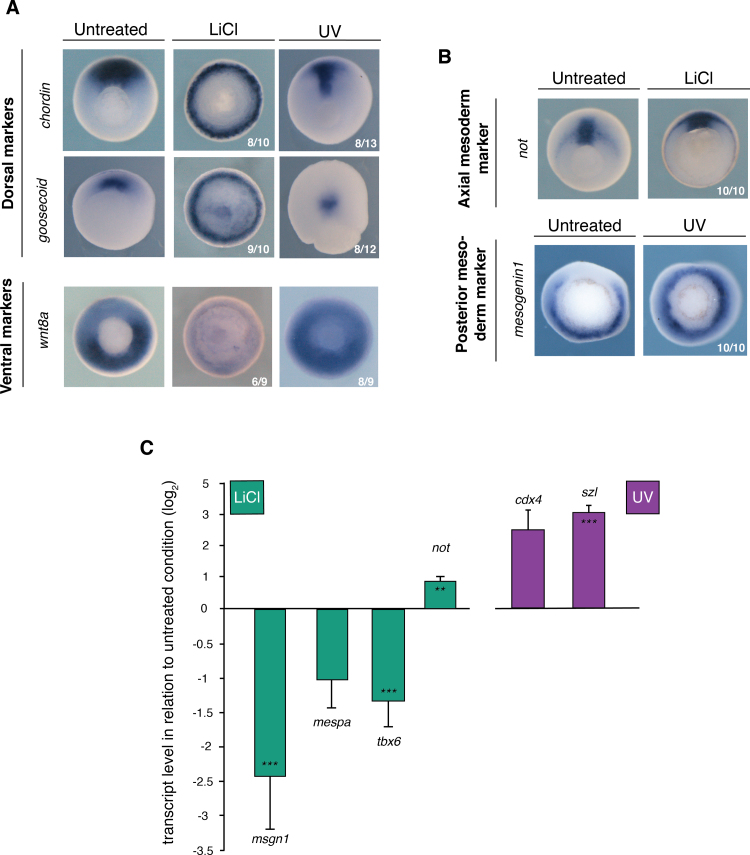
**Verification of UV- and LiCl-induced DV axis perturbation.** (A,B) WMISH of dorsal (*chordin* and *goosecoid*), ventral (*wnt8a*) and mesoderm markers (*not* and *mesogenin1* [*msgn1*]) under indicated conditions at mid-gastrula stage. (C) RT-qPCR of several mesodermal markers under indicated conditions in mid-gastrula embryos. Error bars, SEM of biological triplicates. Student's two-tailed *t*-test: *, p < 0.1; **, p < 0.05; and ***, p < 0.01.

LiCl treatment had a high penetrance with 95–100% (e.g., 49 out of 50) of embryos being hyperdorsalised, while UV irradiation caused 85–95% (e.g., 61 out of 67) of the embryos being hyperventralised. In addition to the DAI score, the treatment efficacies were validated by analysing the expression levels of known dorsal (*gsc, chordin, noggin* and *not*) and ventral (*wnt8a, bmp4* and *msgn1)* markers (Fig. 1B,C and Fig. S1). Expression of dorsal markers expanded ventrally in LiCl-treated embryos while being substantially reduced under UV treatment. Conversely, the expression levels of ventral markers increased in UV-treated embryos and decreased in LiCl-treated embryos.

### 2.2 Transcriptomics of UV or LiCl treatment

While the morphological and some transcriptional effects of UV or LiCl treatment have been well studied, there has been no systematic comparison of the transcriptomes of such embryos. Therefore, poly-A enriched transcriptomes of untreated, LiCl- and UV-treated embryos were analysed for differential expression and enrichment of gene function. For each condition, mid-gastrula embryos (stage 11–11.5) from five independent experiments were profiled, so as to provide statistical power to allow for transcript level variability between clutches and differing efficacies of UV or LiCl treatment. For each biological replicate, some sibling embryos were maintained to the tailbud stage, and only clutches that showed high phenotype penetrance (≥85%) were subjected to total RNA extraction, poly(A) RNA-Seq library generation and deep paired-end sequencing. Results of aligning sequencing reads to gene models v7.2 of the *X. tropicalis* genome assembly v7.1 are summarised in Table S1.

**Fig. 2:**
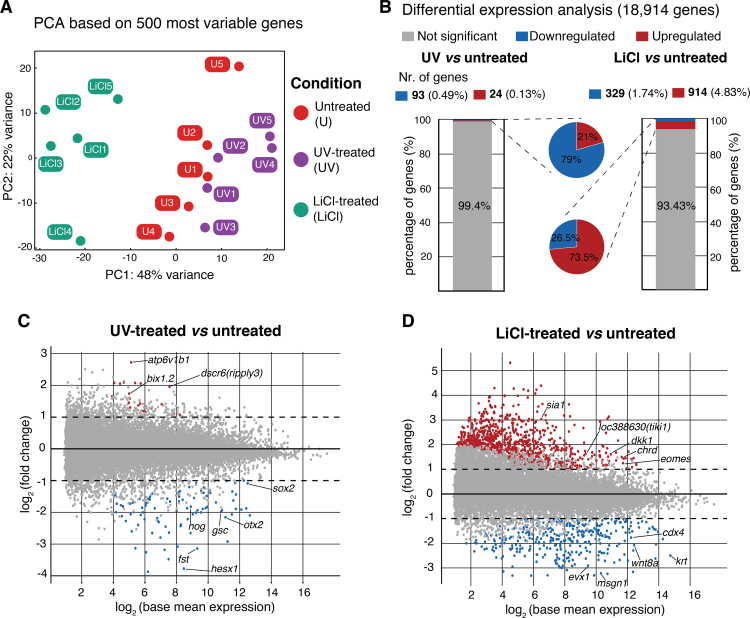
**UV-treated embryos are transcriptionally more closely related to unperturbed embryos than LiCl-treated embryos.** (A) Principal component analysis (PCA) of five biological replicates for each condition. (B-D) Identification of affected genes: transcriptional fold change ≥ 2 with FDR ≤ 1%. (C,D) MA plots of transcriptional fold changes against base mean expressions between indicated conditions.

Overall, principal component analysis (PCA) suggested that the majority of observed variance reflected treatment-induced changes, while the second principal component revealed some variance that separated biological replicates (Fig. 2A). The transcriptome of LiCl-treated embryos was significantly different from that of untreated embryos, and it was slightly more different from that of UV-treated embryos. By contrast, UV treatment did not induce any large-scale shifts of gene expression by the mid-gastrula stage (Fig. 2A). Pairwise comparison with the normal transcriptome corroborated these relationships: LiCl-treated embryos had ten times more significant (at least two-fold change at FDR ≤ 1%) gene expression changes (~ 6.6%) than did UV-treated embryos (~ 0.6%) (Fig. 2B-D). Of the genes whose expression were affected in LiCl-treated embryos, almost 75% were upregulated (914 out of 1243 genes), while genes that were affected by UV treatment were predominantly downregulated (93 out of 117 genes) (Fig. 2B-D).

**Fig. 3:**
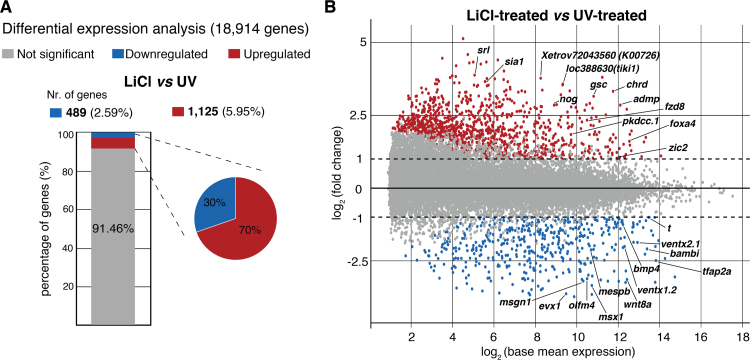
**Sensitive transcriptomics of DV axis determination.** (A) Differential expression analysis between LiCl- and UV-treated embryos. (B) MA plots of transcriptional fold changes against base mean expressions between indicated conditions. Identification of affected genes: transcriptional fold change ≥ 2 with FDR ≤ 1%.

UV and LiCl have opposing effects on development, so to identify genes that are differentially expressed along the DV axis we made a direct comparison of gene expression following the two treatments. About 8.6% of the genes analysed showed significantly different transcript levels (at least two-fold change at FDR ≤ 1%), between UV and LiCl, 70% of which were elevated upon LiCl treatment (Fig. 3A; Table S2 and S3). Among the genes with the highest upregulation in LiCl-treated embryos were early dorsal fate determinants expressed in the Spemann organiser: the homeobox genes *siamois 1 (sia1)* (Lemaire et al., 1995) and *goosecoid (gsc)* (Laurent et al., 1997); BMP antagonists *noggin* (*nog*) (Kessler et al., 1997) and *chordin* (*chrd*) (Piccolo et al., 1999); canonical Wnt regulators *frizzled 8 (fzd8)* (Itoh et al., 1998) and *loc3888630* (*tiki1*) (Zhang et al., 2012); and the secreted tyrosine kinase *pkdcc.1* implicated in morphogenetic movements and planar cell polarity (Ding et al., 2016; Popov et al., 2017; Vitorino et al., 2015). Similar transcriptional stimulation was observed for later markers of dorsal axial and paraxial cell specification, including the neural transcription factor *zic2*, the muscle-specific calcium ion regulator *sarcalumenin* (*srl*), and the chordamesoderm and notochord marker *foxa4* (Fig. 3B).

By contrast, among the 485 genes significantly upregulated in UV-treated embryos were markers of ventro-posterior and non-neural ectoderm development. The former included both ligands (*bmp4* and *wnt8a*) and tissue-specific transcription factors (*evx1*, *msx1*, *mespb, msgn1*, *ventx1.1* and *ventx1.2*) (Fig. 3B).

These observations indicate that LiCl and UV treatment had the expected effect on transcription, regulating the choice between dorso-anterior and ventro-posterior specification, respectively.

### 2.3 UV- and LiCl-induced perturbations show DV-related gene signatures

**Fig. 4:**
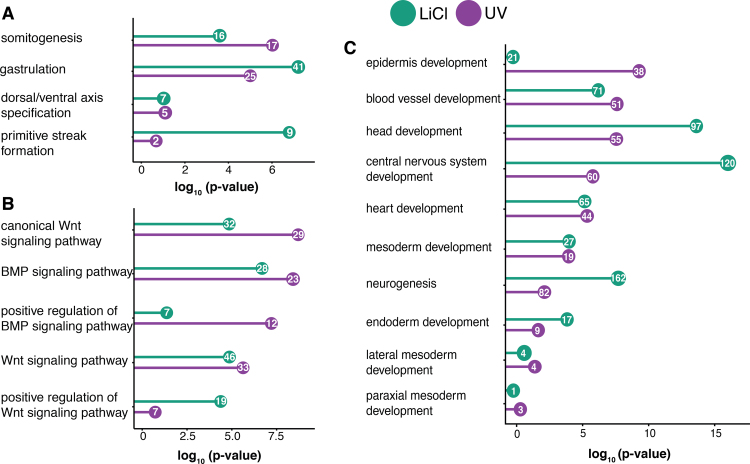
**LiCl and UV treatments affect DV axis determination and neural induction.** Plots show the number of upregulated genes and associated hypergeometric p-values of enrichment for specific biological processes (BPs) of the Gene Ontology (GO) project. Selected BPs refer to early developmental processes (A), signalling (B) and differentiation of germ layers and their derivatives (C).

To characterise the transcriptomes of UV- and LiCl-treated embryos in detail, differentially expressed genes were analysed for enriched biological processes (BPs) of the Gene Ontology (GO) project (Fig. 4). LiCl-triggered gene upregulations were preferentially enriched for the BPs of gastrulation and primitive streak formation (known as blastopore lip formation in *Xenopus*) (Fig. 4A). For example, these BPs were associated with the upregulation of *gsc*, *cer1*, *nodal*, *otx2*, *otx1*, *lhx1* and *hhex* upon LiCl treatment. By contrast, the BP of somitogenesis was overrepresented among upregulated genes in UV-treated embryos, many of which were markers of paraxial mesoderm such as *msgn1*, *mesp2*, *mespA*, *cdx4* and *t*. The enrichment level of genes associated with DV axis specification was similar among upregulated genes in UV- and LiCl-treated embryos.

Many of the genes whose expression were changed in UV- and LiCl-treated embryos were involved in the BMP and Wnt signalling pathways (Fig. 4B). This was consistent with the known effects of these pathways on DV specification. The BMP-related genes overrepresented in UV-treated embryos encode several BMP ligands (*bmp4*, *bmp7.1*) as well as several transcription factors (TFs) of the helix-loop-helix (*hes3.3*, *hes5.1*, *hes6.1* and *hes8*), ventx (*ventx1.1*, *ventx1.2* and *ventx3.2*) family and some other families (*msx1* and *foxj1*). Interpretation of changes in expression of components of the Wnt signalling pathway was more complicated because Wnt signalling normally occurs in two waves in the early embryo: an early maternal wave at the dorsal side and a later zygotic wave, predominantly mediated by Wnt8a, on the ventro-lateral side of the embryo. Upregulated genes in LiCl-treated embryos associated with positive regulation of the pathway included *admp*, *hhex* and the TF family of *zic* genes (*zic1–4*).

At later stages, genes overrepresented in LiCl-treated embryos included those involved in neurogenesis, head development (e.g. *chrd*) and central nervous system development (e.g. *zic* TFs and the blimp-associated TF *prdm12*). Genes overexpressed in UV-treated embryos included those involved in epidermis development (e.g. *keratin* and *grainyhead like TF 3*). Endoderm development was slightly enriched in LiCl-treated embryos (e.g. Nodal ligand *nodal* and TFs *hhex*, *otx2*, *foxa2* and *nkx2-1*). This was consistent with reports that lithium ions potentiate mesendoderm formation in response to Nodal signalling (Cooke and Smith, 1987, Cooke et al., 1989). Expression of genes related to mesoderm formation did not differ between UV- and LiCl-treated embryos.

**Fig. S2:**
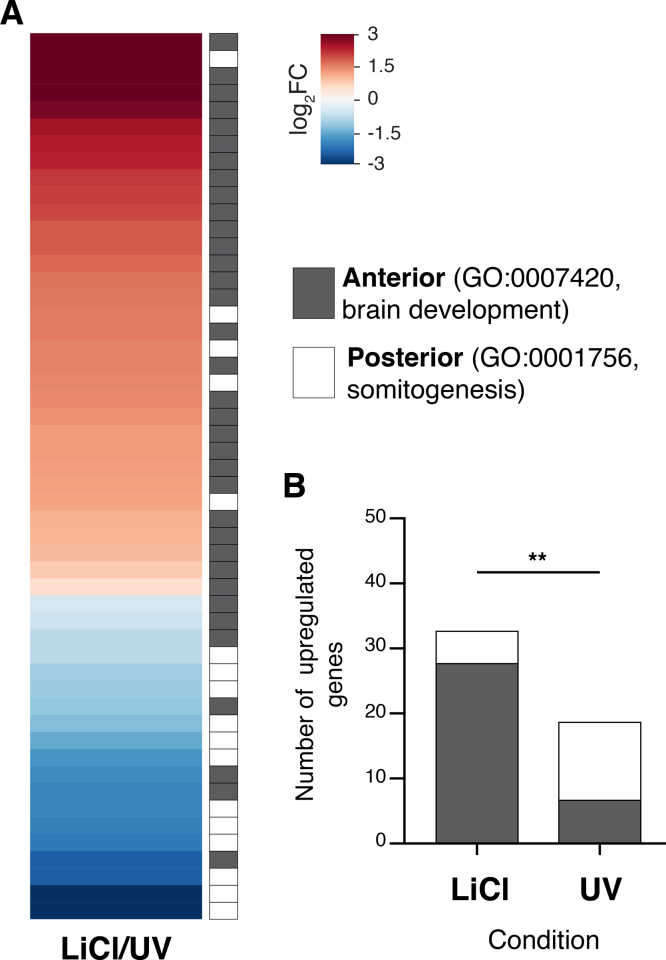
**Linking LiCl/UV-induced transcriptional changes to antero-posterior development.** (A) Heatmap of transcriptional fold changes between LiCl- and UV-treated embryos of genes associated with anterior development (brain) and posterior (somitogenesis) development. (B) Stacked bar graph showing the number of genes that are upregulated in either LiCl- or UV-treated embryos and the GO term associated with the gene. Fisher’s exact test (p = 0.007) suggests that genes upregulated in LiCl- or UV-treated embryos influence anterior and posterior development, respectively.

Although there is no GO term describing anterior or posterior identity we observed that genes involved in anterior (brain) or posterior (somitogenesis) development are upregulated in LiCl- or UV-treated embryos respectively (Fisher's exact test, p-value 0.007) (Fig. S2). This association is consistent with the fate map of the *Xenopus* early gastrula, where prospective anterior and dorsal structures overlap, as do ventral and posterior structures (Lane and Sheets, 2000, Martinez Arias and Steventon, 2018).

### 2.4 DV axis perturbation versus manual dissection of DV tissue

**Fig. 5:**
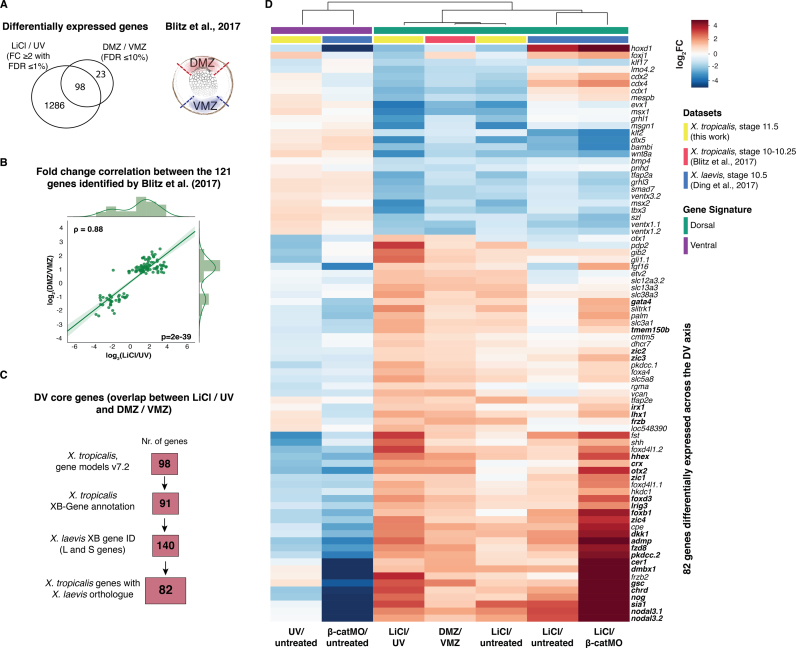
**Comparison with differential gene expression across DV axis confirms that LiCl and UV treatments generate dorso-neuralised and ventro-epidermalised embryos**. (A) Venn diagram of differential gene expression between LiCl and UV (LiCl vs UV, stage 11.5) and across DV axis (DMZ vs VMZ, stage 10–10.25; Blitz et al., 2017). (B) Regression plot of indicated transcriptional fold changes for differential gene expression across the DV axis (Blitz et al., 2017). Pearson coefficient, ρ = 0.88. Student's two-tailed *t*-test, p = 2e-39. (C) Procedure to find *X. laevis* orthologues of the *X. tropicalis* DV core genes via Xenbase (XB) gene identities. L and S refer to gene's location on either the long or short chromosome. If both S and L genes were present, the latter were selected since the L subgenome has been shown to better resemble the ancestral condition (Session et al., 2016). (D) Heatmap of transcriptional fold changes (FC) of DV core genes between indicated conditions. Gene names in bold are part of the early dorsal β-catenin gene signature (Ding et al., 2017).

In an effort to verify our results we compared data from UV- and LiCl-treated embryos with the transcriptional profiles of dorsal and ventral marginal zone (DMZ and VMZ) tissue from untreated embryos (Blitz et al., 2017) (Fig. 5A).

Our pairwise comparison of DMZ and VMZ transcriptomes identified 121 differentially expressed genes (FDR ≤10%), of which 86 were upregulated in DMZ and 35 in VMZ (Fig. 5A,B). Manual dissections thus identified significantly fewer differentially expressed genes than did our UV and LiCl perturbations (1384 genes), but we note that the ratio of upregulated genes in DMZ (~ 71%) versus VMZ (~ 29%) resembles the ratio of upregulated genes in LiCl- versus UV-treated embryos (~ 70% versus ~ 30%). The difference in numbers may derive from the different experimental approaches; the different stages of analysis (stage 10–10.25 for dissection versus st. 11.5 in our experiments); and the number of biological replicates (two for dissection versus five in our experiments).

**Fig. S3:**
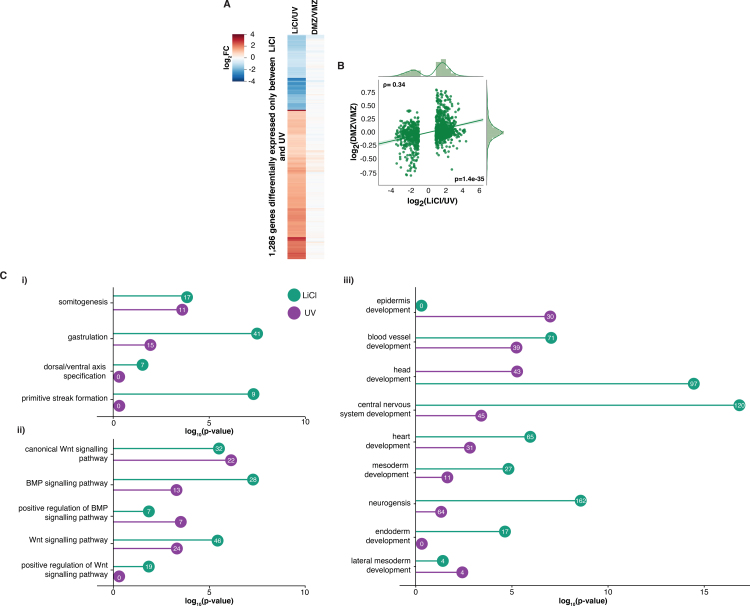
**Gene set enrichment analysis of differentially expressed genes between LiCl- and UV-treated embryos not captured by the comparative transcriptomics of DMZ and VMZ**. (A-C) Analysis of genes differentially expressed between LiCl- and UV-treated embryos, but not captured by the transcriptional comparison of DMZ and VMZ. Heatmap (A) and regression plot (B) of indicated transcriptional comparisons. (B) Pearson coefficient, ρ = 0.34. Student’s two-tailed *t*-test, p = 1.4e-35. (C) Plots show the number of upregulated genes and associated hypergeometric p-values of enrichment for specific biological processes (BPs) of the Gene Ontology (GO) project. Selected BPs refer to early developmental processes (i), signalling (ii), and differentiation of germ layers and their derivatives (iii).

About 80% (98 out of 121) of the genes whose expression differed between DMZ and VMZ regions had differences in transcript level of at least two-fold between LiCl- and UV-treated embryos. Pairwise correlation revealed that in most cases (117 out of 121) where a gene was upregulated in DMZ or VMZ regions, upregulation was also observed in LiCl- or UV-treated embryos, respectively (Fig. 5B,D). The discrepancies (e.g., *myf5*) might derive from differences in experimental approaches, in that dissected DMZs might have contained BMP-exposed lateral marginal zone (LMZ) by the stage of analysis (de Souza et al., 1999, Hopwood et al., 1991; Kim et al., 1998; Wilm and Solnica-Krezel, 2005). We studied transcriptional fold changes between DMZ and VMZ of the 1286 genes identified as differentially expressed only between LiCl- and UV-treated embryos (Fig. S3A). There was a weakly positive correlation between the transcriptional fold changes of the two experiments (Fig. S3B). Some of the genes were involved in DV axis determination while others reflected UV- and LiCl-induced perturbations also affecting antero-posterior patterning and neural induction (Fig. S3C).

**Fig. S4:**
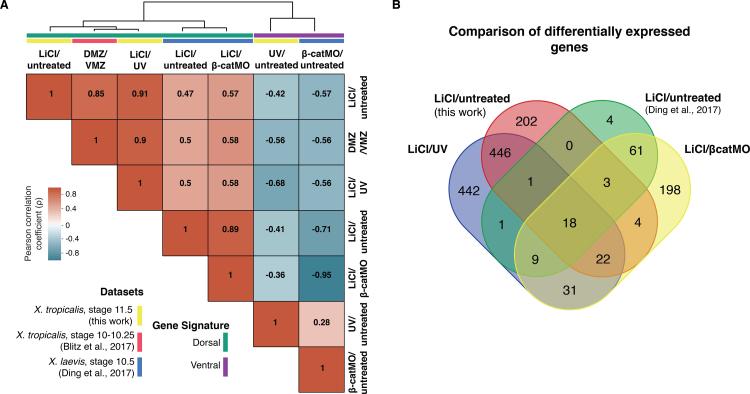
**Comparative transcriptomics of DV axis determination.** (A) Pairwise Pearson correlations of indicated transcriptional comparisons of the DV core genes. This includes work from Blitz et al. (2017) and Ding et al., 2017a, Ding et al., 2017b. Conditions were clustered according to Euclidean distances. (B) Venn diagram comparing genes identified as differentially expressed between indicated conditions (transcriptional fold change ≥ 2 with FDR ≤ 1%). LiCl/untreated (stage 11.5), LiCl/UV (stage 11.5), LiCl/untreated (stage 10.5; Ding et al., 2017) and LiCl/βcatMO (stage 10.5; Ding et al., 2017).

Ding et al., 2017a, Ding et al., 2017b recently defined a dorsal and β-catenin-dependent gene signature for *X. laevis* embryos at early gastrula stage. This was based on the integrative analysis of various transcriptomes from embryos treated with LiCl, embryos injected with β-catenin morpholino (β-catMO), as well as embryos bisected into dorsal and ventral halves. To compare this data with our results, we first defined a core set of 98 DV genes based on the overlapping differential expression between LiCl/UV and DMZ/VMZ in *X. tropicalis* (Fig. 5A,C). This core set was reduced to 82 genes for which orthologues between the two *Xenopus* species and experiments could be found (Fig. 5C). Despite the lower Pearson correlation between *X. laevis* and *X. tropicalis* experiments (Fig. S4A), the transcriptional behaviour of most DV core genes was in line with the treatment and consistent between *Xenopus* species (Fig. 5D). Some inconsistencies of expression level between experiments might be explained by the differences in stage, species, embryo manipulation and library preparation. The genes with highest correlation among all the experiments were immediate-early targets of maternal β-catenin such as *nodal 3.1*, *nodal 3.2* and *sia1* (Fig. 5D).

### 2.5 Validation and characterisation of novel DV markers identified by axis perturbation

To validate our transcriptome dataset, we selected two genes that were upregulated in LiCl-treated embryos (*cpe* and *K00726*) and two that were upregulated in response to UV radiation (*Xetrov72022004* and *c8orf4*). More uncharacterised genes identified by DV axis perturbation are listed in Table S4. The *cpe* gene has recently been identified as part of the dorsal gene signature in *X. laevis* (Ding et al., 2016).

**Fig. 6:**
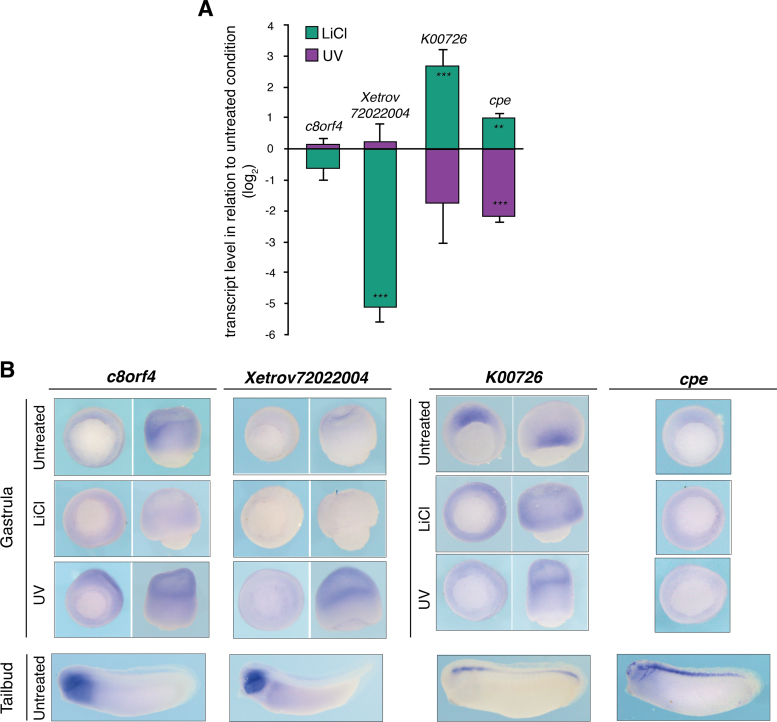
**Expression analysis of uncharacterised genes identified by the means of nongenetic axis perturbation and comparative transcriptomics**. RT-qPCR (A) and WMISH (B) of *c8orf4*, *Xetrov72022004*, *K00726* and *cpe*. (A) Error bars, SEM of biological triplicates. Student's two-tailed *t*-test: *, p < 0.1; **, p < 0.05; and ***, p < 0.01. (B) Mid-gastrula stages, vegetal and lateral view except for *cpe* with vegetal view only; and mid-tailbud stage, lateral view.

Quantitative transcript profiling at the gastrula stage confirmed that *cpe* and *K00726* were upregulated in LiCl-treated embryos and downregulated in UV-treated embryos, while *Xetrov72022004* and *c8orf4* were downregulated in LiCl-treated embryos and unaffected in UV-treated embryos (Fig. 6A). Whole mount in situ hybridisation revealed that *cpe* and *K00726* were expressed in the DMZ at gastrula stages and in the neural tube by tailbud stages. As expected, their expression expanded around the blastopore in LiCl-treated embryos and was reduced in UV-treated embryos. Expression of both *Xetrov72022004* and *c8orf4* was weak and ectodermal in unperturbed embryos (Fig. 6B). By the tailbud stage, expression of both was restricted to the head ectoderm. LiCl treatment significantly reduced their ectodermal expression, consistent with its ability to promote dorsal and neural structures.

### 2.6 β-catenin activates dorsal marker *K00726*, a Rnf220-like ubiquitin ligase

**Fig. 7:**
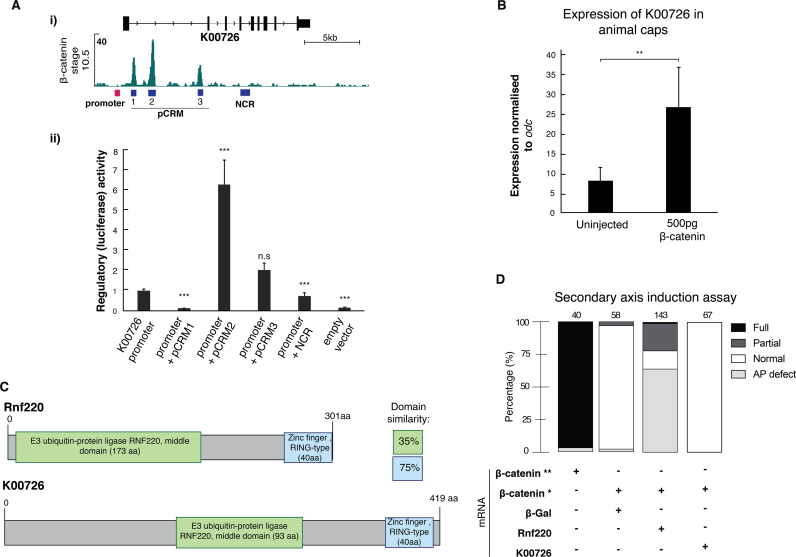
**RNF220-like ubiquitin ligase*****K00726*****is a β-catenin-responsive dorsal marker.** (A) (i) Genomic snapshot of DNA occupancy of β-catenin (Nakamura et al., 2016) at the *K00726* locus in early gastrula embryos. (ii) Luciferase assay for indicated constructs containing *K00726* promoter, putative *cis*-regulatory modules (pCRM) 1–3 or negative control region (NCR). Genomic position of these genomic elements are shown in (i). Reporter activity was normalised to basal activity of the *K00726* promoter. Error bars, SEM of biological duplicates. Student's two-tailed *t*-test: ***, p < 0.01; n.s, not significant. (B) RT-qPCR of *K00726* in animal caps derived from embryos injected with and without 500 pg of *β-catenin* mRNA. Error bars, SEM of biological triplicates. Student's two-tailed *t*-test: **, p < 0.05. (C) Representation of Rnf220 and K00726 protein including known domains. Comparison of the domain sequences revealed that the E3 ubiquitin ligase and zinc-finger RING-type domain have 35% and 75% conservation, respectively. (D) Morphological outcomes of the secondary axis induction assay: AP defect, smaller or kinked antero-posterior axis; partial, secondary axis formed without cement gland; full, secondary axis formed with cement gland. Amount of injected mRNA: β-catenin**, 250–500 pg; β-catenin*, 5–25 pg (subthreshold); *β-Gal*, *K00726* and *Rnf220*, 500–1000 pg. At the top of each bar is the total number of analysed embryos.

The spatial expression pattern of *K00726*, and its response to UV and LiCl treatment, suggested that it might be regulated by Wnt signalling. With this in mind, we noted that chromatin profiling of early gastrula embryos (Nakamura et al., 2016) revealed that β-catenin binds three putative *cis*-regulatory modules within the first intron of *K00726* (pCRM1, pCRM2 and pCRM3) (Fig. 7A.i). The transcriptional activities of the three pCRMs, and a negative control region (NCR), were assayed individually at the early gastrula stage in a dual luciferase reporter assay using the endogenous promoter of *K00726*. The luciferase assay showed that the promoter by itself was capable of increasing reporter expression six-fold compared to a promoterless luciferase vector (Fig. 7A.ii). Addition of pCRM2 or pCRM3 both enhanced promoter activity, with pCRM2, which showed the highest level of β-catenin DNA occupancy, increasing luciferase activity six-fold (Fig. 7A.ii). Interestingly, pCRM1 completely repressed the promoter, while the NCR had no effect. Consistent with these observations overexpression of β-catenin enhanced the transcription of *K00726* in animal caps cultured until the start of gastrulation (Fig. 7B).

K00726 protein shared sequence similarity with Rnf220, a protein that may stabilise β-catenin via its C-terminal RING-type domain, in an interaction mediated by the ubiquitin-specific protease 7 (USP7) (Ma et al., 2014). The conservation between Rnf220 and K00726 is relatively high within the zinc finger RING-type domain (75%), but low within the E3 ubiquitin protein ligase domain (35%) (Fig. 7C). With this in mind, we asked whether K00726 could enhance Wnt signalling. We used an assay based on that of Ma et al. (2014), in which subthreshold amounts of β-catenin mRNA, incapable of inducing a secondary axis, were co-injected with mouse *Rnf220* or *K00726* mRNA into ventral blastomeres of *X. laevis*. In contrast to Rnf220, K00726 was unable to induce a secondary axis or to cause any other axial defects (Fig. 7D).

## 3 Discussion

Amphibian embryos provide a powerful model system for the study of early vertebrate development because they are available in large numbers and because they are so easy to manipulate. Here, we use the classic techniques of UV and LiCl treatment to enrich the *X. tropicalis* embryo for ventral or dorsal cell types, and we then explore the transcriptomes of these embryos in an effort to discover new genes involved in the specification of DV asymmetry.

While both UV- and LiCl-treated embryos display gastrulation defects, only the transcriptome of LiCl-treated embryos deviates substantially from that of unperturbed embryos. This is at least in part because UV-treated embryos lack only the rather small dorsal organiser region, whereas the marginal zone of LiCl-treated embryos consists almost entirely of dorsal organiser cells at the expense of any lateral and ventral cell types. However, the transcriptome-wide comparison of LiCl- and UV-treated embryos also indicates that the treatments significantly affect the binary choice of ectodermal cells towards epidermis or neural tissue. UV-treated embryos fail to produce BMP antagonists in the DMZ and consequently, dorsally expanded BMP signals both ventralise the marginal zone and generate an excess of epidermis at the expense of neural tissue, creating an ‘aneural’ phenotype (Scharf and Gerhart, 1980). For the most part, our results are in line with previous transcriptome-wide studies exploring DV asymmetry by manual dissections or axis perturbation (Blitz et al., 2017, Ding et al., 2017a, Ding et al., 2017b)

Our transcriptomics of DV perturbation has identified low-expression genes whose transcript levels are increased upon LiCl (*cpe* and *K00726*) or UV (*Xetrov72022004* and *c8orf4*) treatment. Further experiments suggest that *K00726*, a Rnf220-like ubiquitin ligase, forms part of a β-catenin-dependent early dorsal gene signature in *Xenopus*. We have identified two β-catenin^+^ *cis*-regulatory modules in the first intron of *K00726* that are capable of significantly enhancing transcription.

Overall, our analysis suggests that perturbation-mediated cell type enrichment provides a powerful technique to reveal rare transcripts differentially expressed across specific body axes. We believe the datasets generated in this work will be useful for researchers studying DV axis determination. The differential expression of such genes between LiCl- and UV-treated embryos may predict their spatial expression and their contributions to cell fate decisions.

## 4 Material and methods

### 4.1 Embryo culture and manipulation

All animal procedures were performed under licence as required by the Animals (Scientific Procedures) Act 1986 (UK). Standard procedures were used for *Xenopus* ovulation (Khokha et al., 2002, Sive et al., 2007). *X. tropicalis* and *X. laevis* eggs were fertilised in vitro. The embryos were cultured in 0.05x Marc's Modified Ringer's solution (MMR) at 25–28 °C or 0.1x Normal Amphibian Medium (NAM) at 14–20 °C, respectively. Approximately 10 min after fertilisation, embryos were dejellied with 2.2% [w/v] L-cysteine diluted in the appropriate culture medium (pH 8.0). Injections were carried out with *X. laevis* embryos equilibrated in 4% Ficoll PM-400 (Sigma)/0.1x NAM. Injected embryos were transferred to 0.1x NAM without Ficoll once they reached about stage 8. Embryos were staged according to Nieuwkoop and Faber (1994).

#### 4.1.1 UV irradiation

Immediately after removing their jelly coats, 50–100×*X. tropicalis* fertilised eggs were transferred into a 50-ml conical centrifuge tube filled to the top with 0.05x MMR. The tube was closed by wrapping Saran cling film around the top using rubber bands and turned upside down allowing the embryos to settle on the film. The tube was swirled gently to position the zygotes to the centre of the film wrapping. The tube was placed onto a styrofoam hole whose depth of 2 cm kept vegetal poles at the appropriate distance from a compact UV lamp (UVP, UVGL-25). The embryos were irradiated with 254 nm shortwave UV for 2 min. After that, the tube was kept upside down and transferred to the incubator without disturbing the embryos until the completion of the first cell division. The embryos were then transferred to an agarose-coated petri dish containing 0.05x MMR and cultured to the developmental stage of interest.

#### 4.1.2 LiCl treatment

At the 32-cell stage, dejellied *X. tropicalis* embryos were transferred to an agarose-coated petri dish containing 0.3 M LiCl/0.05x MMR. They were gently swirled in the dish and incubated for 5 min. LiCl treatment was terminated by rinsing the embryos in 0.05x MMR five times. The embryos were then cultured in 0.05x MMR to the desired developmental stage.

#### 4.1.3 Microinjection

A total volume of 10 nl of mRNA and/or plasmid DNA was injected into *X. laevis* embryos at the 1-cell, 2-cell or 4-cell stage using a microinjector (Narishige IM-300). Microinjection needles were produced from borosilicate glass capillaries (Harvard Apparatus, GC120-15) using a micropipette puller (Sutter p97).

#### 4.1.4 Animal caps

Approximately 500 pg of *β-catenin* mRNA was injected into the animal side of each blastomere of *X. laevis* 2-cell embryos. At stage 8, embryos were transferred into 0.7x MMR and animal caps were cut using 13 µm wire tip electrodes (Protech International, 13-Y1) at medium voltage (setting 2 on the MC-2010 micro cautery instrument, Protech International). After dissection, caps were transferred to a fresh agarose-coated plate containing 0.7x MMR and incubated at 18 °C alongside whole sibling embryos until stage 15. Animal caps were processed for total RNA extraction.

### 4.2 Cloning

Forward and reverse primers were designed based on the genomic sequence of *X. tropicalis* (assembly v7.1) to clone *cpe*, *Xetrov72022004*, *K00726* and *c8orf4* from gastrula-staged cDNA (see Table S5). GoTaq-amplified gene fragments were cloned into pGEM-T Easy vector (Promega).

For luciferase assays, the *K00726* promoter, three putative *cis*-regulatory modules (pCRM1–3), and a negative control region (NCR) were amplified from *X. tropicalis* genomic DNA using KAPA HiFi HotStart ReadyMix (KAPA Biosystems) and reverse and forward primers containing 5′ *Hind*III (promoter) or *Bam*HI (enhancer) restriction sites (Table S5). The *K00726* promoter was inserted into the *Hind*III site of promoterless pGL3-Basic vector. Single pCRMs were inserted into the *Bam*HI site of the pGL3 vector equipped with the endogenous promoter.

### 4.3 Whole mount in situ hybridisation

Whole mount in situ hybridisation (WMISH) was carried out as described (Monsoro-Burq, 2007) with digoxigenin-labelled RNA probes. Antisense (AS) probes were generated from linearised cDNA clones in vitro with the appropriate RNA polymerase (Roche) and digoxigenin-11-UTP. The following AS probes were transcribed with T7 RNA polymerase from plasmids linearised with *Eco*RI unless otherwise specified: pGEM-5Zf(-)[*noggin*] (W. C. Smith and Harland, 1992); pBS-SK(-)[*goosecoid*] (Cho et al., 1991); pBS-SK(-)[*chordin*] (Sasai et al., 1994); pGEM1[*wnt8a*] linearised with *Bam*HI (Christian et al., 1991); pBS-SK(+)[*bmp4*]; pCS2(+)[*not1*] linearised with *Xho*I (Dassow et al., 1993); pCMV-Sport6.ccdB[*msgn1*] (Gentsch et al., 2013); pGEM-T[*c8orf4*] linearised with *Nde*I; pGEM-T[*Xetrov72022004*] linearised with *Sac*I; pGEM-T[*K00726*] linearised with *Sac*I; and pGEM-T[*cpe*] linearised with *Apa*I and transcribed with SP6 RNA polymerase. After DNase treatment, probes were precipitated with LiCl overnight at − 20 °C and rinsed with 80% EtOH. The precipitate was dissolved with RNase-free water. The WMISH probes were diluted with hybridisation buffer (50% [v/v] formamide, 5x SSC, 1x Denhardt's, 10 mM EDTA, 1 mg/ml torula RNA, 100 μg/ml heparin, 0.1% [v/v] Tween-20% and 0.1% [w/v] CHAPS) to a final concentration of 10 ng/µl (10x stock).

### 4.4 Synthesis of capped mRNA for microinjection

Approximately 1 μg of linearised (with *Not*I unless otherwise specified) expression vector was transcribed using the mMESSAGE mMACHINE SP6 Transcription Kit (Thermo Fisher Scientific): pCS2^+^[3xHA-*K00726*] linearised with *Apa*I; pCS2^+^[*β-catenin-*GFP] (Addgene, #16839); pSP64T[n*lacZ*] (nuclear β-galactosidase); pCS2^+^[*rluc*] (*Renilla* luciferase) (Demagny et al., 2014); and pCS2^+^[*mRNF220*-FLAG].

### 4.5 Total RNA extraction and cDNA synthesis

10–20 embryos or 15–20 animal caps were homogenised in 200 µl of TRIzol (Thermo Fisher Scientific) by vortexing for 10 min at room temperature. The homogenate was mixed with 40 µl of chloroform and centrifuged for 5 min at 4 °C at full speed. The upper phase was transferred to a clean 1.5-ml Eppendorf tube. RNA was precipitated using the RNA Zymo Clean and Concentrator-5 or -25 kit (Zymo Research). Approximately 1 μg of total RNA was converted into first strand cDNA using the SuperScript III reverse transcriptase (Thermo Fisher Scientific).

### 4.6 Quantification of transcript levels

Gene expression levels were determined by absolute quantification using SYBR Green I reagents (Roche) and the LightCycler LC480 II (Roche). Each cDNA sample was run in technical triplicates. The standard curve for each primer pair was calculated from eight crossing points (C_t_-values) reflecting amplifications from a 1:3 serial dilution of wild-type cDNA. The primer sequences are listed in Table S5. Microsoft Excel was used to normalise quantifications to the housekeeping gene *odc1*, design plots and perform paired Student's two-tailed *t*-tests.

### 4.7 Library preparation, sequencing and biocomputational analysis

Total RNA representing 10 *X. tropicalis* embryos (15–20 μg) was processed according to the low sample protocol of the TruSeq RNA sample preparation guide version 2 (Illumina). The concentration of the library was determined by fluorometry using Qubit dsDNA HS reagents (Thermo Fisher Scientific). The quality of the library was verified by micro-capillary electrophoresis using the Agilent 2100 Bioanalyzer. Libraries were read paired-end along 50 bases on the HiSeq. 2500 machine (Illumina). The reads were mapped to the *X. tropicalis* gene models v7.2 using the splice-aware aligner Tophat2 (D. Kim et al., 2013) disabling any search for novel splice junctions. Reads were counted per gene using the python script HTSeq-count in ‘intersection-nonempty’ mode (Anders et al., 2015). The raw read counts from each experiment were merged before running the R package DESeq. 2 (Love et al., 2014) to determine differential gene expression between selected conditions (Table S6). The gene set enrichment analysis was carried out using previously assembled GO annotations (Gentsch et al., 2015) and the R package GOstats (Falcon and Gentleman, 2007). All sequencing reads and raw read counts are available at the Gene Expression Omnibus (GEO) database under the accession number GSE107424.

### 4.8 Dual luciferase reporter assay

Embryos were injected with 50 pg of various firefly luciferase reporter constructs (pGL3-Basic, pGL3[*K00726* promoter], pGL3[*K00726* promoter + pCRM1], pGL3[*K00726* promoter + pCRM2], pGL3[*K00726* promoter + pCRM3], pGL3[*K00726* promoter + NCR]) and 5 pg of *Renilla luciferase* mRNA. Embryos were harvested at early gastrula stage 10. We used the reagents of the Dual-Luciferase Reporter Assay System kit (Promega) to perform the luciferase assay. Embryos were homogenised in 100 µl of 1xPassive Lysis Buffer. The homogenate was spun for 1 min at 16,000 g and 4 °C. 20 µl of the supernatant was transferred to 96-well plates (solid bottom, white). 50 µl of LARII was added to the supernatant. Firefly luminescence was measured twice for each sample with a Perkin Elmer ENVision 2102 Multilabel plate reader. After these readings, 50 µl of 1x Stop&Glo reagent was added and *Renilla* luminescence was recorded twice for each sample. The *Renilla* signal was used to normalise firefly measurements.

## Appendix A Supplementary material

Supplementary materialTable S1**. Summary of RNA-Seq read alignments.** Paired-end reads were aligned to the *X. tropicalis* gene models v7.2.

Supplementary materialTable S2**. Upregulated genes in LiCl-treated embryos at gastrula stage.** The list includes genes with transcriptional fold changes ≥ 2 (FDR ≤ 1%).

Supplementary materialTable S3**. Upregulated genes in UV-treated embryos at gastrula stage.** The list includes genes with transcriptional fold changes ≥ 2 (FDR ≤ 1%).

Supplementary materialTable S4**. Shortlist of mostly uncharacterised genes upregulated upon UV or LiCl treatment.** The list includes expression data from the *X. tropicalis* gene expression database (http://genomics.crick.ac.uk/apps/profiles/) (Collart et al., 2014, Owens et al., 2016) and further details from Xenbase (http://www.xenbase.org) (Karpinka et al., 2015).

Supplementary materialTable S5. List **of primer sequences for cloning and RT-qPCR.**

Supplementary materialTable S6**. Unfiltered output of differential gene expression analysis between LiCl- and UV-treated embryos using DESeq2.**
