# Supplementary material for: Transcriptomics of dorso-ventral axis determination in Xenopus tropicalis
Source: Dev Biol. 2018 Jul 15;439(2):69–79. doi: 10.1016/j.ydbio.2018.04.022 (PMC5971218; doi:10.1016/j.ydbio.2018.04.022)
Supplement: Supplementary file 4 — Supplementary materialTable S4. Shortlist of mostly uncharacterised genes upregulated upon UV or LiCl treatment. The list includes expression data from the X. tropicalis gene expression database (http://genomics.crick.ac.uk/apps/profiles/) (Collart et al., 2014, Owens et al., 2016) and further details from Xenbase (http://www.xenbase.org) (Karpinka et al., 2015). [file mmc8.docx]

| Gene | Name | Condition | Expression | Function/Information |
| --- | --- | --- | --- | --- |
| *cpe* | *CarboxypeptidaseE* | LiCl | Small peak of expression just before stage 12 and increases at later stages. | It is a zinc carboxypeptidase: Catalysis the release of C-terminal arginine or lysine from polypeptides. Part of the dorsal signature identified in (Ding et al., 2017). |
| *f3* | *Coagulin factor III* | LiCl | Maternally expressed, peaks at stage NF10 | N/A |
| *K00726* | *N/A* | LiCl | Starts to be expressed at the start of gastrulation, peaks around stage 15 and decreases from then on. | There is no data published data about the gene. Contains an E3 ubiquitin-ligase RNF220 domain, giving it high homology to the protein rnf220, a positive regulator of Wnt signalling. |
| *mmp17* | *Matrix metallopeptidase 17* | LiCl | Expression peaks at stage NF10 | N/A |
| *nedd9* | *Neural precursor cell expressed developmentally down-regulated 9* | LiCl | Expression starts at MBT | Signal transducing adaptor protein |
| *pgm2l1* | *Phosphoglucomutase 2-like 1* | LiCl | Maternal transcript whose levels peak at stage NF9. Reached lowest level at stage NF12 | N/A |
| *ptpru* | *Protein tyrosinase phosphatase, receptor u* | LiCl | Expression starts at stage NF10, dorsally. Later it is expressed in the floorplate, brain and pronopheric kidney. | Ectopic expression prevents Wnt-mediated posteriorization of the neural plate (Morgan et al., 2003) |
| *srl* | *sarcalumenin* | LiCl | Expressed after stage NF14, in the somites. | No literature referring specifically to the gene. It interacts with apobec2. |
| *tdgf1p2* | *Teratocarcinoma-derived growth factor 1, member 2. A.K.A:xcr2* | LiCl | Expressed at stage NF10 and peaks at stage NF16. |  |
| *tmem150b* | *Transmembrane protein 150b* | LiCl | Transcription starts at stage NF9, peaking at NF12. Expressed in the endoderm and mesendoderm. | Identified as part of the dorsal  signature in *Xenopus* embryos (Ding et al., 2017). |
| *Xetrov72018683* | *LOC100124853* | LiCl | Starts to be expressed at stage NF10. | Predicted to be FRAS1 related extracellular matrix 1 (frem1), transcript variant X3. However, the gene frem1 is annotated elsewhere in the genome. |
| *Xetrov72021302* | *N/A* | LiCl | Maternally expressed, peaks at stage NF10. | Predicted to be hematopoietic SH2 domain containing. |
| *Xetrov72027551* | *unnamed* | LiCl | Expression peaks at stage NF10 and at stage NF15 | Predicted to be insulin-2 like LOC100494294. |
| *Xetrov72037173* | *N/A* | LiCl | Expressed at low levels high variability between clutches. | Contains a EGF-like module containing, mucin-like, hormone receptor-like sequence. Similar to emr-1, an adhesion G-protein coupled receptor |
| *bspry* | *B-box and SPRY domain containing* | UV | Highly expressed during gastrula stages | N/A |
| *c8orf4* | *Chromosome 8 open reading frame 4* | UV | Starts at MBT increasing during gastrulation and  peaks at stage 14, increasing again at stage 32. | Also known as TC-1, a transposable element associated  with neural tissue (Faunes et al., 2011). |
| *dip2a* | *disco interacting protein 2 homolog A* | UV | Maternally expressed and peaks at stage NF9  and then decreases during gastrulation,  recovering the levels at stage NF15 | In humans has been shown to bind to FSTL1 (follistatin like 1)  and in the mouse it is expressed in the  visceral mesoderm (Ouchi et al., 2010). |
| *fam83a* | *family with sequence similarity 83 member A* | UV | Expression starts during gastrulation at low levels | N/A |
| *ptger4* | *prostaglandin E receptor 4* | UV | Highly expressed during gastrula stages, peaking at stage NF12. | N/A |
| *slc35d2* | *solute carrier family 35 (UDP-GlcNAc/UDP-glucose transporter), member D2* | UV | Maternally expressed and peaks at stage NF9 and then decreases during gastrulation. | In *Drosophila* has been suggested to be involved in the regulation of nucleotide-sugar levels that can differently  affect Wingless and two distinct aspects of Notch signalling (Goto et al., 2001). |
| *sh3d21* | *SH3 domain containing 21* | UV | Expressed during gastrulation | Adaptor protein CMS/SETA. |
| *styk1* | *serine/threonine/tyrosine kinase 1* | UV | Transcribed at stage NF10 and peaks at stage NF12. | Known to activate MAP kinase and Pl3k |
| *vgll1* | *vestigial like family member 1* | UV | Expressed after gastrulation |  |
| *Xetrov72002596* | *N/A* | UV | Expressed during gastrulation | BLAST resulting in one hit - serine/threonine protein kinase N2-like (LOC105948381). |
| *Xetrov72003455* | *N/A* | UV | Highly expressed during gastrula stages | N/A |
| *Xetrov72022004* | *N/A* | UV | Expression starts at stage 10 and peaks just after stage 12, decreasing during tailbud stages. | N/A |
| *Xetrov72027348* | *LOC100495414* | UV | Highly expressed during gastrula stages, peaking at stage NF12. | Predicted to be Hes-5-like (LOC100495414). |

**References:**

Ding, Y., Colozza, G., Zhang, K., Moriyama, Y., Ploper, D., Sosa, E.A., Benitez, M.D.J., De Robertis, E.M., 2017. Genome-wide analysis of dorsal and ventral transcriptomes of the Xenopus laevis gastrula. Developmental Biology 426, 176–187.

Faunes, F., Sánchez, N., Moreno, M., Olivares, G.H., Lee-Liu, D., Almonacid, L., Slater, A.W., Norambuena, T., Taft, R.J., Mattick, J.S., Melo, F., Larrain, J., 2011. Expression of Transposable Elements in Neural Tissues during Xenopus Development. PLoS ONE 6, e22569.

Goto, S., Taniguchi, M., Muraoka, M., Toyoda, H., Sado, Y., Kawakita, M., Hayashi, S., 2001. UDP|[ndash]|sugar transporter implicated in glycosylation and processing of Notch. Nat. Cell Biol. 3, 816–822.

Morgan, R., El-Kadi, A.-M., Theokli, C., 2003. Flamingo, a cadherin-type receptor involved in the Drosophila planar polarity pathway, can block signaling via the canonical wnt pathway in Xenopus laevis. Int. J. Dev. Biol. 47, 245–252.

Ouchi, N., Asaumi, Y., Ohashi, K., Higuchi, A., Sono-Romanelli, S., Oshima, Y., Walsh, K., 2010. DIP2A functions as a FSTL1 receptor. J. Biol. Chem. 285, 7127–7134.
